# Genetic Variants in *KIR/HLA-C* Genes Are Associated With the Susceptibility to HCV Infection in a High-Risk Chinese Population

**DOI:** 10.3389/fimmu.2021.632353

**Published:** 2021-06-18

**Authors:** Chao Shen, Zhijun Ge, Chen Dong, Chunhui Wang, Jianguo Shao, Weihua Cai, Peng Huang, Haozhi Fan, Jun Li, Yun Zhang, Ming Yue

**Affiliations:** ^1^ Department of Epidemiology and Biostatistics, Key Laboratory of Infectious Diseases, School of Public Health, Nanjing Medical University, Nanjing, China; ^2^ Department of Critical Care Medicine, The Affiliated Yixing Hospital of Jiangsu University, Yixing, China; ^3^ Department of Epidemiology and Statistics, School of Public Health, Medical College of Soochow University, Suzhou, China; ^4^ Institute of Epidemiology and Microbiology, Eastern Theater Command Centers for Disease Control and Prevention, Nanjing, China; ^5^ Department of Epidemiology, Center for Global Health, School of Public Health, Nanjing Medical University, Nanjing, China; ^6^ Department of Digestive Medicine, Third Affiliated Hospital of Nantong University, Nantong, China; ^7^ Department of General Surgery, Third Affiliated Hospital of Nantong University, Nantong, China; ^8^ Department of Information, First Affiliated Hospital of Nanjing Medical University, Nanjing, China; ^9^ Department of Infectious Diseases, First Affiliated Hospital of Nanjing Medical University, Nanjing, China

**Keywords:** hepatitis C virus, killer cell immunoglobulin-like receptors, polymorphism, human leukocyte antigen, infection

## Abstract

**Background:**

KIR/HLA-C signaling pathway influences the innate immune response which is the first defense to hepatitis C virus (HCV) infection. The aim of this study was to determine the association between the genetic polymorphisms of *KIR/HLA-C* genes and the outcomes of HCV infection in a high-risk Chinese population.

**Methods:**

In this case-control study, four single nucleotide polymorphisms (SNPs) of *KIR/HLA-C* genes (*KIR2DS4*/*KIR2DS1*/*KIR2DL1* rs35440472, *HLA-C* rs2308557, *HLA-C* rs1130838, and *HLA-C* rs2524094) were genotyped by TaqMan assay among drug users and hemodialysis (HD) patients including 1,378 uninfected control cases, 307 subjects with spontaneous viral clearance, and 217 patients with persistent HCV infection. Bioinformatics analysis was used to functionally annotate the SNPs.

**Results:**

After logistic regression analysis, the rs35440472-A and rs1130838-A alleles were found to be associated with a significantly elevated risk of HCV infection (OR = 1.562, 95% CI: 1.229–1.987, *P* < 0.001; OR = 2.134, 95% CI: 1.180–3.858, *P* = 0.012, respectively), which remained significant after Bonferroni correction (0.05/4). The combined effect of their risk alleles and risk genotypes (rs35440472-AA and rs1130838-AA) were linked to the increased risk of HCV infection in a locus-dosage manner (all *P*_trend_ < 0.001). Based on the SNPinfo web server, rs35440472 was predicted to be a transcription factor binding site (TFBS) while rs1130838 was predicted to have a splicing (ESE or ESS) function.

**Conclusion:**

*KIR2DS4*/*KIR2DS1*/*KIR2DL1* rs35440472-A and *HLA-C* rs1130838-A variants are associated with increased susceptibility to HCV infection in a high-risk Chinese population.

## Introduction

Hepatitis C virus (HCV) infection is a global health problem, affecting more than 185 million individuals worldwide (1, 2). Among them, approximately 71 million people have progressed to chronic HCV infection and over 10 million are in China (3). Without effective and timely treatment, chronic HCV infection can induce decompensated cirrhosis and hepatocellular carcinoma (HCC), resulting in significant socioeconomic burden (4). Despite the high cure rates of direct-acting antivirals (DAAs), HCV elimination continues to be a challenge due to frequent re-infection in the high-risk population (5). Elucidating the mechanisms underlying HCV infection and development is essential to conducting precision prevention for the high-risk population and eliminating HCV.

The initiation and progression of HCV infection depend on a complex interplay of pathogenic and host factors. As we all know, immune responses affect disease development and innate immunity is the first line of defense against HCV. As a crucial member of the innate immune system, natural killer cells (NK cells), are well known for their rapid and efficient immune responses against HCV infection (6, 7). Furthermore, the functional activation of NK cells can affect HCV RNA replication as well as the expression of HCV proteins (8). The NK cell response and host susceptibility to infection are tightly regulated by specific immune receptors and their corresponding ligands (9–11).

Killer cell immunoglobulin-like receptors (KIRs), an important receptor of NK cells, are type I transmembrane glycoproteins belonging to the immunoglobulin superfamily (12). KIRs can activate or inhibit NK cells by recognizing class I major histocompatibility complex (MHC) molecules (13–15). Generally, the nucleated cells normally express inhibitory ligands, such as human leukocyte antigen class I (HLA-I), on their surface that keep the NK cells in an inactivated state by engaging with the KIRs (7). In contrast, virus-infected cells might reduce the expression of MHC molecules and present activating ligands on their surfaces. NK cells would be elicited and exert their antiviral effector functions to participate in immune responses (16, 17). However, aberrant activation of NK cells resulting in excessive or low immune responses may lead to persistent infection (18, 19). Not surprisingly, the exertion of NK cell function is directly impacted by the genetic variations of KIRs and HLA-I molecules (12, 20).

The *KIR* gene cluster is located on chromosome 19q13.4 within the 150 kb long leukocyte receptor complex region (21, 22). HLA-I proteins are encoded by the MHC genes that span a region of about 3.6 Mb on chromosome 6p21.3 (23, 24). The independent segregation of unlinked *KIR* and *HLA-I* genes both feature significant variations, which could affect the health and disease status in different individuals (25). Recent studies have shown that the diversity in the number and type of *KIR-HLA* gene combinations are associated with infections, cancer, and reproductive disorders (26–29). Dring et al. reported an association between *KIR2DS3* and chronic HCV infection, and Nozawa et al. found that the presence of *KIR2DL3* and *HLA-C1* was associated with both treatment-induced and spontaneous clearance of HCV infection in the Caucasian population (30, 31). Recently, exciting researches have shown that KIR2DS4 selectively binds to HLA-C (32). This finding may be important for the host NK cell response and the outcome of viral infection, including the susceptibility to HIV and hepatitis B virus-associated hepatocellular carcinoma (33, 34). It is interesting that rs35440472 is attributed to *KIR2DS4*, *KIR2DS1*, and *KIR2DL1* according to the NCBI dbSNP. *KIR2DS4/KIR2DS1/KIR2DL1* rs35440472 was abbreviated as *KIR2DS4* rs35440472 when appropriate in this study. In addition, even a single nucleotide polymorphism (SNP) also can markedly alter KIRs expression, and the specificity and activity of HLA-I (12, 35–38). However, little is known regarding the association between *KIR/HLA-C* SNPs and the outcome of HCV infection. Given all of the above, we hypothesized that SNPs of these signaling pathway genes may influence the outcomes of HCV infection. Therefore, the current study was to explore the associations between four potentially functional SNPs in the genes of the *KIR/HLA-C* and outcomes of HCV infection in a high-risk Chinese population, including *KIR2DS4*/*KIR2DS1*/*KIR2DL1* rs35440472 G>A, *HLA-C* rs1130838 G>A, *HLA-C* rs2524094 A>G, and *HLA-C* rs2308557 A>G.

## Method

### Ethics Statement

This study was conducted according to the national legislation and the ethical guidelines of the Declaration of Helsinki (39), and approved by the Institutional Review Board of Nanjing Medical University. Written consent was obtained from all individuals before participating in this study and sampling.

### Subjects

A total of 1,902 high-risk subjects, including 1,153 drug users from two compulsory detoxification centers (Nanjing and Yixing, China) and 749 hemodialysis (HD) patients from nine hospital-based hemodialysis centers (Jiangsu, China), were consecutively recruited from 2008 to 2018. The exclusion criteria were as follows (1): age below 18 years or over 80 years, (2) history of any anti-HCV treatment, (3) co-infection with human immunodeficiency virus (HIV) or other hepatitis viruses, and (4) any autoimmune, alcoholic, or metabolic liver diseases. The participants were categorized into the following groups: (1) Group A—uninfected controls who were seronegative for both anti-HCV antibodies and HCV RNA; (2) Group B—spontaneous HCV clearance, seropositive for anti-HCV antibodies and seronegative for HCV RNA; (3) Group C—persistent HCV infection and seropositive for both anti-HCV antibodies and HCV RNA. All subjects came from high-risk populations and had high-risk behaviors. Therefore, all three groups were exposed to HCV. The serological tests were conducted at least three times during the 6-month follow-up period to confirm the infection status. The demographic data and history of HCV exposure were recorded by interviewing each participant with a standard questionnaire.

### Serological Testing

Blood samples (5–10 ml) were collected from all subjects and centrifuged to separate the plasma fractions, which were then stored at −20°C. Anti-HCV antibodies were detected through the qualitative third-generation enzyme-linked immunosorbent assay (ELISA) (Diagnostic Kit for Antibody to HCV 3.0 ELISA, Intec Products Inc, Xiamen, China) which is a qualitative experiment. Trizol LS reagent (Takara Biotech, Tokyo, Japan) was used to extract HCV RNA from the sera. The HCV RNA load was measured by real time fluorescent quantitative polymerase chain reaction with the detection limit of 1 × 10^3 IU/ml (Promotor^®^ Hepatitis C Virus Quantitative PCR Test Kits; ACON Biotech Co., Ltd., Hangzhou, China). HCV genotypes were determined using the murex HCV serotype ELISA kit (Abbott, Wiesbaden, Germany) based on type-specific antibodies. Genomic DNA was extracted from the white blood cells (WBCs) using protease K digestion, followed by phenol-chloroform purification and ethanol precipitation. All serological tests were performed with the same analytical systems.

### SNP Selection and Genotyping

The selection of candidate genes is based on the importance and the frequencies of *KIR/HLA-C* genes among the Chinese population. Candidate Tag SNPs of these genes were selected by searching the 1000 Genomes database (37.0 version, https://www.internationalgenome.org/home) and the HaploReg database Version 4.1 (http://pubs.broadinstitute.org/mammals/haploreg/haploreg.php). The selected SNPs were filtered using the following criteria: (1) minor allele frequency (MAF) >5% in the Chinese Han population and (2) pairwise linkage disequilibrium (LD) r^2^ ≥ 0.8. The above data can be acquired from Haploview software (version 4.2; Broad Institute, Cambridge, MA, USA). The immune-related *KIR2DS4*/*HLA-C* SNPs reported in previous studies were also considered. Finally, four SNP candidates (*KIR2DS4*/*KIR2DS1*/*KIR2DL1* rs35440472, *HLA-C* rs2308557, *HLA-C* rs1130838, and *HLA-C* rs2524094) were chosen for genotyping. It should be mentioned that, according to the NCBI dbSNP, rs35440472 is shown to belong to *KIR2DS4*, *KIR2DS1*, and *KIR2DL1*. Not surprisingly, rs35440472 is located in the overlapping region of three genes. Rs35440472 is presented in these three genes, which all encode receptors for HLA-C. For simplicity, *KIR2DS4/KIR2DS1/KIR2DL1* rs35440472 was abbreviated as *KIR2DS4* rs35440472.

Genotyping of the four SNPs was conducted with a TaqMan allelic discrimination assay on the LightCycler^®^ 480 IIReal-Time PCR System (Roche, Switzerland). The primers and probes are summarized in **Table S1**. The SNPs were genotyped in a blinded manner, and the success rate was >90%. Furthermore, 10% random samples were re-genotyped and the accordance rates were 100%. Besides, all tests were carried out in accordance with the manufacturer’s instructions. The genotyping experiments of all samples used identical procedures and instruments.

### *In Silico* Analysis

The functions of the selected SNPs were predicted using the HaploReg database Version 4.1 (https://pubs.broadinstitute.org/mammals/haploreg/haploreg.php) and the SNPinfo Web Server (https://snpinfo.niehs.nih.gov/cgi-bin/snpinfo/snpfunc.cgi). The RNAfold Web Server (http://rna.tbi.univie.ac.at//cgi-bin/RNAWebSuite/RNAfold.cgi) was applied to detect mRNA secondary structures. The expression quantitative trait loci (eQTL) analysis was conducted with the Genotype-Tissue Expression (GTEx) database (https://www.gtexportal.org/home/snp/). The UCSC Genome Browser (http://genome.ucsc.edu/) was used to assess the potential biological function. The H3K4Me1 histone marker expression data in the GM12878, H1-hESC, HSMM, HUVEC, K562, NHEK, NHLF, and HepG2 cell lines were analyzed with the Encyclopedia of DNA Elements (ENCODE).

### Statistical Analysis

The demographic, clinical, virological data were compared using the chi-square (*χ^2^*) test, one-way analysis of variance (ANOVA), or Kruskal-Wallis test as appropriate. Hardy-Weinberg equilibrium (HWE) was assessed in the control group by goodness-of-fit *χ^2^* test. The co-dominant model, dominant, additive, and recessive genetic models were used to explore the association between each SNP and the outcomes of HCV infection. Logistic regression adjusted for gender, age, ALT, AST, *IL28B*-rs12979860, *IL28B-*rs8099917, and route of infection was performed to analyze the relationship between these SNPs and HCV infection outcome by calculating odds ratio (OR) and 95% confidence interval (CI). Stratified analysis was used to further reduce the bias of confounding factors. All statistical analyses were two-sided with a significance level of *P* < 0.05 was considered statistically significant. Bonferroni correction was applied to correct for multiple comparisons and the *P*-value was adjusted to 0.0125 (0.05/4) (40). All statistical analyses were performed using SPSS (version 22.0; SPSS Institute, Chicago, IL, USA) and Stata (version14.0; STATA Corp, College Station, TX, USA).

## Results

### Demographic and Clinical Characteristics

The demographic and clinical characteristics of all subjects are summarized in **Table 1**. Group A consisted of 1,378 individuals (1,003 males and 375 females; mean age 42.92 ± 12.91 years), Group B included 307 subjects (218 males and 89 females; mean age 41.61 ± 13.10 years), and Group C included 217 cases (154 males and 63 females; mean age 39.44 ± 11.25 years). There were no significant differences in the distribution of gender and the frequency of *IL28B*-rs12979860 among the three groups (*P* = 0.737 and *P* = 0.439, respectively). However, age, alanine aminotransferase (ALT), aspartate aminotransferase (AST), routes of infection, HCV genotype, and the frequency of *IL28B*-rs8099917 showed significant differences (all *P* < 0.001). Furthermore, the demographic and clinical characteristics of the HD and IVDU patients are shown in **Tables S1** and **S2**, respectively. The genotype distributions in the control group were in accordance with the Hardy-Weinberg Equilibrium for all six SNPs (all *P*>0.05) (**Table S3**).

**Table 1:** Demographic and clinical characteristics among HCV control, spontaneous clearance, and persistent infection groups.

Variables	Group A (%)	Group B (%)	Group C (%)	*P*
	n = 1378	n = 307	n = 217	
Age (mean ± SD)	42.92 ± 12.91	41.61 ± 13.10	39.44 ± 11.25	<0.001^a^
Gender				0.737
Male	1,003 (72.79)	218 (71.01)	154 (70.97)	
Female	375 (27.21)	89 (28.99)	63 (29.03)	
ALT (U/L)				<0.001^b^
<40	1,209 (88.57)	221 (71.99)	147 (67.74)	
≥40	156 (11.43)	86 (28.01)	70 (32.26)	
AST (U/L)				
<40	1,294 (94.87)	242 (80.94)	158 (75.96)	<0.001^b^
≥40	70 (5.13)	57 (19.06)	50 (24.04)	
Routes of infection				<0.001^b^
HD	581 (42.16)	92 (29.97)	76 (35.02)	
IVDU	797 (57.84)	215 (70.03)	141 (64.98)	
HCV genotype				<0.001^b^
1	–	42 (27.27)	136 (63.26)	
Non-1	–	73 (47.40)	31 (14.42)	
Mixed	–	39 (25.32)	48 (22.33)	
*IL28B*-rs12979860				0.439^b^
CC	1163 (84.46)	263 (85.95)	190 (87.56)	
CT/TT	214 (15.54)	43 (14.05)	27 (12.44)	
*IL28B*-rs8099917				<0.001^b^
TT	988 (75.02)	259 (84.36)	191 (88.43)	
TG/GG	329 (24.98)	48 (15.64)	25 (11.57)	

Group A: uninfected control cases; Group B: spontaneous clearance subjects; Group C: persistent infection patients.

HCV, hepatitis C virus; SD, standard deviation; ALT, alanine transaminase; AST, aspartate transaminase; HD, hemodialysis patients; IVDU, Intravenous drug user.

Non-1 means genotype 2 and 3; Mixed means genotype1/2, 1/3, 2/3, and 1/2/3.

a P value of Kruskal-Wallis test among three/two groups.

b P value of χ^2^-test among three/two groups.

### Association of Candidate SNPs With the Susceptibility and Chronicity of HCV Infection

The genotype distribution of rs35440472, rs1130838, rs2524094, and rs2308557 in the three groups is shown in **Table 2**. A representative TaqMan allelic discrimination assay result of rs35440472 is shown in **Figure S1**. To analyze the association between these SNPs and susceptibility to HCV infection, Group B and Group C patients were combined into the HCV-infected group and compared them with the control Group A. After adjusting for gender, age, ALT, AST, *IL28B*-rs12979860, *IL28B*-rs8099917, and route of infection, the results of logistic regression analysis revealed that *KIR2DS4-*rs35440472 and *HLA-C*-rs1130838 were associated with susceptibility to HCV infection. Furthermore, the frequency of the rs35440472-A allele was significantly higher in the infected *versus* the uninfected groups compared to that of the wild type rs35440472-G allele (dominant model: adjusted OR = 1.562, 95% CI = 1.229–1.987, *P* < 0.001; recessive model: adjusted OR = 1.460, 95% CI = 1.108–1.924, *P* = 0.007; additive model: adjusted OR = 1.361, 95% CI = 1.165–1.589, *P* < 0.001). Compared to rs1130838-GG genotype, the AA genotype correlated to a significantly increased risk of HCV infection (co-dominant model: adjusted OR = 2.134, 95% CI = 1.180–3.858, *P* = 0.012). Furthermore, the association between the selected SNPs and HCV infection chronicity was compared among the persistent infection subjects (Group C) and the spontaneous infection subjects (Group B). However, no significant association was observed in the logistic regression analyses between these four SNPs and HCV infection chronicity (all *P* > 0.05/4) (**Table 2**).

**Table 2:** Genotypes distributions of *KIR/HLA-C* genes among HCV control, spontaneous clearance, and persistent infection groups.

SNPs (genotype)	Group A n (%)	Group B n (%)	Group C n (%)	Group B+C n (%)	OR (95% CI)^a^	*P* ^a^	OR (95% CI)^b^	*P* ^b^
	n = 1,378	n = 307	n = 217	n = 524				
rs35440472						0.000		0.204
GG	479 (38.23)	97 (31.60)	57 (26.27)	154 (29.39)	1.00	–	1.00	–
GA	568 (45.33)	136 (44.30)	113 (52.07)	249 (47.52)	**1.464 (1.133–1.892)**	**0.004**	1.430 (0.926–2.209)	0.107
AA	206 (16.44)	74 (24.10)	47 (21.66)	121 (23.09)	**1.820 (1.327–2.496)**	**<0.001**	1.205 (0.720–2.018)	0.478
Dominant model					**1.562 (1.229–1.987)**	**<0.001**	1.352 (0.899–2.033)	0.147
Additive model					**1.361 (1.165–1.589)**	**<0.001**	1.113 (0.863–1.435)	0.410
Recessive model					**1.460 (1.108–1.924)**	**0.007**	0.965 (0.624–1.494)	0.884
rs1130838						0.058		0.130
GG	990 (71.84)	210 (68.40)	155 (71.43)	365 (69.66)	1.00	–	1.00	–
GA	348 (25.25)	86 (28.01)	48 (22.12)	134 (25.57)	1.090 (0.849–1.399)	0.500	0.761 (0.497–1.167)	0.211
AA	40 (2.90)	11 (3.58)	14 (6.45)	25 (4.77)	**2.134 (1.180–3.858)**	**0.012**	2.154 (0.888–5.221)	0.089
Dominant model					1.174 (0.925–1.490)	0.186	0.891 (0.598–1.327)	0.569
Additive model					1.222 (0.998–1.498)	0.053	1.041 (0.752–1.441)	0.811
Recessive model					2.084 (1.157–3.752)	0.014	2.326 (0.967–5.595)	0.059
rs2524094						0.041		0.675
AA	868 (64.68)	175 (57.19)	132 (60.83)	307 (58.70)	1.00	–	1.00	–
AG	409 (30.48)	107 (34.97)	68 (31.34)	175 (33.46)	1.100 (0.867–1.397)	0.431	0.853 (0.572–1.272)	0.434
GG	65 (4.84)	24 (7.84)	17 (7.83)	41 (7.84)	1.593 (1.012–2. 507)	0.044	0.967 (0.466–2.004)	0.927
Dominant model					1.168 (0.933–1.462)	0.176	0.872 (0.599–1.269)	0.474
Additive model					1.182 (0.988–1.414)	0.067	0.922 (0.687–1.238)	0.590
Recessive model					1.543 (0.987–2.412)	0.057	1.020 (0.498–2.090)	0.956
rs2308557					0.143		0.179	
AA	796 (57.85)	200 (65.15)	128 (58.99)	328 (62.60)	1.00	–	1.00	–
AG	479 (34.81)	91 (29.64)	70 (32.68)	161 (30.73)	0.843 (0.664–1.069)	0.158	1.138 (0.761–1.703)	0.529
GG	101 (7.34)	16 (5.21)	19 (8.76)	35 (6.68)	0.736 (0.472–1.149)	0.177	1.735 (0.822–3.660)	0.148
Dominant model					0.823 (0.658–1.028)	0.087	1.226 (0.840–1.789)	0.291
Additive model					0.851 (0.714–1.015)	0.073	1.233 (0.916–1.659)	0.167
Recessive model					0.780 (0.503–1.208)	0.265	1.662 (0.797–3.466)	0.175

KIR, killer cell immunoglobulin-like receptor; HLA-C, human leukocyte antigen class C; SNP, single nucleotide polymorphism; HCV, hepatitis C virus; OR, odds ratio; CI, confidence interval.

Group A: uninfected control cases; Group B: spontaneous clearance subjects; Group C: persistent infection patients; Group (B+C): Infected individuals.

a P value, OR, and 95% CIs of Group (B+C) versus Group A were calculated based on the logistic regression model, adjusted by gender, age, ALT, AST, IL28B-rs12979860, IL28B-rs8099917, and route of infection.

b P value, OR, and 95% CIs of Group C versus Group B were calculated based on the logistic regression model, adjusted by gender, age, ALT, AST, IL28B-rs12979860, IL28B-rs8099917, and route of infection.

Bold type indicates statistically significant results.

A stratified analysis was conducted to decrease the bias of gender, age, ALT, AST, and route of infection. Compared to the GG genotype of rs35440472, a significantly higher of infection was associated with the A allele in the following subgroups: age <50 years (adjusted OR: 1.417, 95% CI: 1.181–1.699, *P*<0.001), the male subgroup (adjusted OR: 1.378, 95% CI: 1.144–1.659, *P* = 0.001), ALT < 40 (adjusted OR: 1.348, 95% CI: 1.135–1.602, *P* = 0.001), ALT ≥ 40 (adjusted OR: 1.503, 95% CI: 1.028–2.197, *P* = 0.035), AST < 40 (adjusted OR: 1.392, 95% CI: 1.180–1.641, *P*<0.001), and IVDU (adjusted OR: 1.399, 95% CI: 1.145–1.710, *P* = 0.008). In addition, rs1130838 variant genotypes were significantly associated with an increased risk of HCV infection in the age ≥50 years (adjusted OR: 4.542, 95% CI: 1.492–13.830, *P* = 0.008), AST < 40 (adjusted OR: 2.397, 95% CI: 1.261–4.557, *P* = 0.008), and HD (adjusted OR: 4.027, 95% CI: 1.534–10.576, *P* = 0.005) subgroups (**Table S4**).

### Combined Effects Analysis

The combined effects of *KIR2DS4* rs35440472 and *HLA-C* rs1130838 on susceptibility to HCV infection were calculated by counting the number of their risk alleles (rs35440472-A and rs1130838-A) and risk genotypes (rs35440472-AA and rs1130838-AA) respectively. The risk for HCV infection increased with the presence of more unfavorable alleles (*P*_Trend_ < 0.001), and carrying all four unfavorable alleles correlated to the highest risk (adjusted OR = 3.629, 95% CI = 1.174–11.213, *P* = 0.025) (**Table 3**). Similarly, the more unfavorable alleles the subjects carried, the more likely they were to be infected with HCV (*P*_Trend_<0.001). Compared with those carrying the rs35440472-GG/GA or rs1130838-GG/GA genotypes, subjects with rs35440472-AA or rs1130838-AA (one risk genotype: adjusted OR = 1.476, 95% CI = 1.120–1.945, *P* = 0.006; two risk genotypes: adjusted OR = 1.793, 95% CI = 1.278–2.516, *P* = 0.001) were more susceptible to HCV infection (**Table 3**).

**Table 3:** The combined effects of risk alleles and genotypes on the risk of HCV infection.

Risk alleles ^a^	Group A	Group (B+C)	HCV-infection	OR (95% CI)^c^	*P^c^*
	n (%)	n (%)	Rate (%)		
0	344 (27.45)	111 (21.18)	24.40	1.00	–
1	528 (42.14)	202 (38.55)	27.67	1.309 (0.978–1.753)	0.070
2	307 (24.50)	167 (31.87)	35.23	**1.920 (1.407–2.619)**	**<0.001**
3	64 (5.11)	37 (7.06)	36.63	1.598 (0.958–2.619)	0.073
4	10 (0.80)	7 (1.34)	41.18	**3.629 (1.174–11.213)**	**0.025**
Trend					<0.001^d^
0	344 (27.67)	111 (21.47)	24.40	1.00	
1–4	899 (72.33)	406 (78.53)	31.11	**1.542 (1.181–2.012)**	**0.001**
Risk genotypes ^b^					
0	344 (27.45)	111 (21.18)	24.40	1.00	–
1	688 (54.91)	297 (56.68)	30.15	**1.476 (1.120–1.945)**	**0.006**
2	221 (17.64)	116 (22.14)	34.42	**1.793 (1.278–2.516)**	**0.001**
Trend					<0.001^d^
0	344 (27.45)	111 (21.18)	24.40	1.00	
1–2	909 (72.55)	413 (78.82)	31.24	**1.553 (1.191–2.024)**	**0.001**

HCV, hepatitis C virus; OR, odds ratio; CI, confidence interval.

Group A: uninfected control cases; Group B: spontaneous clearance subjects; Group C: persistent infection patients; Group (B+C): Infected individuals.

a Number of unfavorable alleles (rs35440472-A and rs1130838-A).

b Number of unfavorable genotypes (rs35440472-AA and rs1130838-AA).

c P value, OR, and 95% CIs of Group A and Group (B+C) were calculated based on the logistic regression model, adjusted by gender, age, ALT, AST, IL28B-rs12979860, IL28B-rs8099917, and route of infection.

d P value for the Cochran-Armitage trend test.

Bold type indicates statistically significant results.

### Bioinformatics Analysis

**Figure 1:**
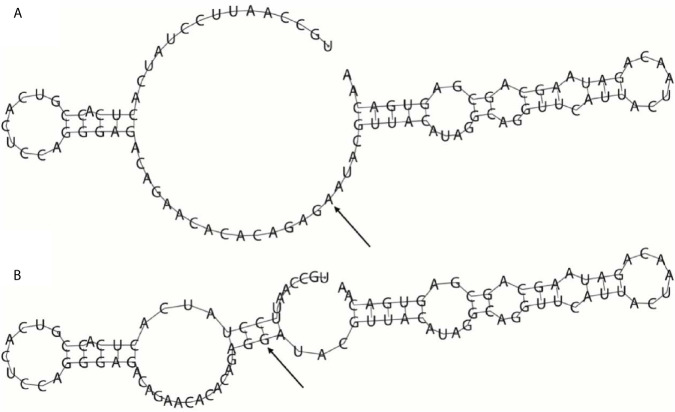
The influence of rs35440472 on KIR2DS4 mRNA optimal secondary structures. **(A)** The minimum free energy of the mRNA optimal secondary structure with a minimum free energy of −10.70 kcal/mol for rs35440472-A; **(B)** The minimum free energy of the mRNA optimal secondary structure with a minimum free energy of −11.70 kcal/mol for rs35440472-G. Changes in the local structure were illustrated by the RNAfold Web Server (Data available: http://rna.tbi.univie.ac.at//cgi-bin/RNAWebSuite/RNAfold.cgi). The arrow marks the position of the mutation (50 bases upstream and 50 bases downstream from the mutation).

Since rs35440472 is located in the overlapping region of *KIR2DS4*, *KIR2DS1*, and *KIR2DL1*, the bioinformatics analysis results can be applied to all three genes. Rs35440472 was predicted to be a transcription factor binding site (TFBS) by using the SNPinfo web server. The RNAfold web server showed the impact of this mutation on the mRNA secondary structure of gene. The local structural changes are shown in **Figure 1**, which indicate a difference in the lowest free energy between rs35440472-A and -G alleles (−10.70 *vs.* −11.70 kcal/mol). Furthermore, the eQTL analysis with the GTEx database showed that the expression of rs35440472-AA was lower than that of rs35440472-AG and GG in the liver tissue (*P* < 0.001) (**Figure S2**).

Based on the HaploReg database, rs1130838 is located in the functional regions of enhancer histone marks, DNase, and PLZF Motifs. In addition, there are 10 SNPs in linkage disequilibrium (r^2^ = 1.0) with rs1130838 in the Asian population (**Table S5**). According to the SNPinfo web server and UCSC, rs1130838 was predicted to have a splicing (ESE or ESS) function and located on the high peak of the histone H3 at lysine 4 (H3K4Me1) marker in seven cell lines as well as in the HepG2 liver cell line from Encyclopedia of DNA Elements (ENCODE) (**Figure S3**).

## Discussion

This study systematically explored the association between SNPs in *KIR/HLA-C* genes and HCV infection. Our results indicate that the rs35440472-A and *HLA-C* rs1130838-A alleles are associated with a significantly higher probability of HCV infection in two high-risk Chinese populations, including drug users and hemodialysis patients. The unfavorable effects of both alleles were also confirmed by stratified analysis. The *KIR/HLA-C* genes may influence NK cell effector functions by regulating signal transduction *via* the activator and inhibitor receptors, which in turn affect the outcomes of HCV infection (41). The KIRs modulate NK cell stimulation through their signaling strength and specificity of interaction. Besides, strongly inhibitory KIR/HLA combinations display a higher threshold for cell activation than those with weak inhibitory action (12).

In this study, the selection of candidate genes is based on the importance of haplotypes of complex *KIR/HLA* genes and the gene frequency in the Chinese population. Therefore, the *KIR2DS4* gene was chosen because it was the only active gene in haplotype A of the *KIR* gene cluster that encodes inhibitory receptors, and it is highly prevalent in the Chinese Han population. The frequency of *KIR2DS4* in the Chinese, Japanese, Caucasian, and African American populations are 92.7, 87, 94.9, and 100%, respectively (25, 42–44). Consistent with this, the frequency of *KIR2DS4* in this study was around 93%. Furthermore, KIR2DS4 binds with multiple HLA ligands encoded by the *HLA-C1*, *HLA-C2*, HLA-A*11:02 (45), and *HLA-F* (46). However, a recent study showed that KIR2DS4 exhibits peptide selective binding to HLA-C which might be significant for the NK cell response to human bacterial infections (32). Apart from bacterial infections, *KIR2DS4* may be associated with the outcome of viral infections. It is associated with susceptibility to HIV and hepatitis B virus-associated hepatocellular carcinoma (33, 34, 47). Therefore, *KIR2DS4* and *HLA-C* were selected as candidate genes, followed by the adoption of a screening strategy to select candidate SNP. In addition, this study is a part of our research series for *KIR/HLA*, and more *KIR/HLA* genes will be explored in further researches.

The rs35440472-A allele (chromosome position: 54846254) in particular was correlated to a higher susceptibility to HCV infection. Interestingly, the NCBI dbSNP shows that rs35440472 belongs to *KIR2DS4*, *KIR2DS1*, and *KIR2DL1*. All of these three genes encode receptors for HLA-C, which also justify this study on HLA-C as a ligand of rs35440472-possessing KIRs. The “multiple attribution” of rs35440472 is reasonable because this site is located in the overlapping region of three genes. It is due to the complex splicing mechanism of KIR gene cluster (48). A kind of splicing event defined as “cryptic exon” is involved. For simplicity, this site is called *KIR2DS4* rs35440472 in most of the text. Rs35440472 doesn’t have a direct protein-coding function because it is located on these three genes’ intron region. Rs35440472 is a potential TFBS with homotypic clusters that are a key component of transcriptional promoters and enhancers (49) and is therefore biologically relevant on basis of the SNPinfo web server. Beyond that, according to predictions about this SNP location in the *KIR2DS4*/*KIR2DS1*/*KIR2DL1* genes, the optimal mRNA secondary structure has changed and the minimum free energy for the A allele is higher than the G allele. The eQTL analysis further revealed that the genetic variation of rs35440472 affected the expression of the *KIR2DS4*/*KIR2DS1*/*KIR2DL1* genes and the rs35440472-A allele reduced the gene expression. Given that KIR2DS4/KIR2DS1 are activating receptors of NK cells (45), rs35440472-A may result in lower activating signals due to decreased gene expression levels. However, not only other activating KIR, KIR2DS1, but also the inhibitory KIR2DL1 possess this SNP and that inhibitory KIRs exert stronger effect than activating ones. Thus, the variation of rs35440472 may affect NK cell effector functions, which ultimately influence the innate immune responses and susceptibility to HCV infection. However, no significant association was found between rs35440472 and the chronicity of HCV infection. These results are consistent with the findings of De Re et al. and Li et al., who also excluded the association between *KIR2DS4* and chronic HCV infection in the Chinese Han or Italian populations, although the grouping was slightly different (50, 51).

The *HLA-C* rs1130838-A allele was also linked to an increased risk of HCV infection. Rs1130838 (chromosome position: 31269347) maps to the exon region of *HLA-C* and its allele changes create missense mutations resulting in residue changes (Thr > Pro, Thr > Ala or Thr > Ser). Studies show that missense mutations are often severely detrimental because they cause complete loss of function (52). Not surprisingly, variations in rs1130838 have been associated with psoriasis and Behcet’s disease (BD) (53). Additionally, rs1130838 has a splicing (ESE or ESS) function and is located on the high peak of the H3K4Me1 marker in different cell lines, including the hepatic carcinoma HepG2 cells. Recent studies show that H3K4me1 affects enhancers by promoting binding of the BAF complex and possibly other chromatin regulators (54, 55). The accumulating evidence implied that the genetic or structural disruption of enhancer function is a major cause of human diseases (56). Therefore, we inferred rs1130838 may affect the susceptibility to HVC by regulating the transcription and translation of *HLA-C*.

In the combined effects analyses of rs35440472 and *HLA-C* rs1130838, it is revealed that the more unfavorable alleles (rs35440472-A and rs1130838-A) or genotypes (rs35440472-AA and rs1130838-AA) patients carried, the more susceptible to HCV they were. The activating receptor KIR2DS4 recognizes unique, selective HLA-C molecules and six HLA-C allotypes (three carrying the C1 epitope and three carrying the C2 epitope) can reliably bind KIR2DS4 (45). The activating KIR2DS4/KIR2DS1 or the inhibitory KIR2DL1, which exerts stronger effect than activating ones, has to interact with HLA-C presenting on healthy or diseased cells to affect NK cell activity (19). As mentioned above, the risk alleles of rs35440472 and rs1130838 could affect the expression of three *KIR* genes as well as *HLA-C*, respectively. Taken together, the variation of these two SNPs not only may affect the activation of KIRs but also the KIR receptors binding with HLA-C ligands because of the incorrect expression of *HLA-C* gene, as the risk alleles increase. Hence, this may be the reason why the SNPs affect NK cell functions, and ultimately associated with the susceptibility to HCV infection. However, the explanations above were based on bioinformatics analysis and further studies are warranted to confirm the functional contribution of rs35440472-A and rs1130838-A in NK cell response to HCV infection.

In our research, some issues need to be mentioned. To minimize inherent selection bias, we adjusted for confounding factors such as gender, age, ALT, AST, and route of infection. Moreover, the *IL28B*-rs12979860 and *IL28B-*rs8099917 were also included because both are significantly associated with both spontaneous virus clearance and response to peg-IFN-α/RBV treatment (57–60). Therefore, the two SNPs were also included as the adjusted factors in logistic regression. However, all subjects came from China and more large survey samples of other nations and ethnic populations are needed to confirm the findings. Regrettably, this study couldn’t extract more data of some subjects including the duration of HD and the number of blood transfusions due to the privacy protection policy.

Nevertheless, there are several limitations in this study that ought to be considered. Firstly, there is a single 22 bp deletion in exon 5 of *KIR2DS4*, which can form a new truncated soluble KIR2DS4 protein. It is defined as KIR1D protein devoid of transmembrane region (45). The effect of KIR1D must be stronger than a presumable effect of SNPs increasing or decreasing receptor expression. And no linkage disequilibrium between the deletion mutation and *KIR2DS4* rs35440472 was found. However, even if it is in LD in KIR2DS4 itself, it cannot generally display LD because it is present also in two other KIRs not possessing this deletion. Since the *KIR2DS4-del* variant may have influenced our findings, the association between KIR2DS4/KIR1D and the outcomes of HCV infection should be explored next. Secondly, KIR2DS4 may bind not only HLA-C, but also HLA-A*11 (45) and HLA-F (61). As HLA-F is only marginally polymorphic and has a limited tissue distribution, it should not play important role here. However, HLA-A is at least as polymorphic as HLA-C, and is generally regarded as more important in antigen recognition by T cells (although much less important for the NK cells). Therefore, the possible association between the genetic polymorphisms within KIR2DS4/HLA-A*11 and the outcomes of HCV infection should also be explored further. Finally, further genetic and functional studies are warranted to assure the mechanism of *KIR/HLA-C* genetic variation in the course of HCV infection.

## Conclusion

In summary, our results revealed that polymorphisms within the *KIR/HLA-C* pathway genes are associated with HCV susceptibility in a high-risk Chinese population. *KIR2DS4*/*KIR2DS1*/*KIR2DL1* rs35440472 and *HLA-C* rs1130838 might serve as potential biomarkers and own potential functions of the risk and progression of HCV infection.

## Data Availability Statement

According to national legislation/guidelines, specifically the Administrative Regulations of the People’s Republic of China on Human Genetic Resources (http://www.gov.cn/zhengce/content/2019-06/10/content_5398829.htm, http://english.www.gov.cn/policies/latest_releases/2019/06/10/content_281476708945462.htm, http://english.www.gov.cn/policies/latest_releases/2019/06/10/content_281476708945462.htm), no additional raw data is available at this time. Data of this project can be accessed after an approval application to the China National Genebank (CNGB, https://db.cngb.org/cnsa/). Please refer to https://db.cngb.org/, or email: CNGBdb@cngb.org for detailed application guidance. The accession code CNP0001926 should be included in the application.

## Ethics Statement

The studies involving human participants were reviewed and approved by the Institutional Ethics Review Committee of Nanjing Medical University (Nanjing, China). The patients/participants provided their written informed consent to participate in this study.

## Author Contributions

CS, MY, and ZG participated in the design of the study. CS, ZG, WC, JS, PH, and WC carried out the surveys and experiments. CS, HF, and CD performed the statistical analysis. YZ, JL, and MY contributed to analysis. CS, ZG, and MY wrote the paper. All authors contributed to the article and approved the submitted version.

## Funding

This study was supported by Science Foundation for Distinguished Young Scholars of Jiangsu Province (BK20190106), the National Natural Science Foundation of China (81773499), Clinical Research Center for emerging respiratory infectious diseases (HS2020002), Key Project of Natural Science Foundation of Yunnan Province (2019FA005), Jiangsu Program for Young Medical Talents (QNRC2016616), and Natural Science Foundation of Jiangsu Province (No. BK20171054).

## Conflict of Interest

The authors declare that the research was conducted in the absence of any commercial or financial relationships that could be construed as a potential conflict of interest.
