# Supplementary material for: Genetic Variants in KIR/HLA-C Genes Are Associated With the Susceptibility to HCV Infection in a High-Risk Chinese Population
Source: Front Immunol. 2021 Jun 18;12:632353. doi: 10.3389/fimmu.2021.632353 (PMC8253047; doi:10.3389/fimmu.2021.632353)
Supplement: Supplementary file 1 [file DataSheet_1.doc]

Supplementary Material

# Supplementary Figures and Tables

## Supplementary Tables

**Supplementary Table 1. Demographic and clinical characteristics among HCV control, spontaneous clearance and persistent infection groups in HD patients.**

| Variables | Group A (%) | Group B (%) | Group C (%) | *P* |
| --- | --- | --- | --- | --- |
|  | n=581 | n=92 | n=76 |  |
| Age (mean ± SD) | 51.24 ± 13.11 | 55.57 ± 11.47 | 49.61 ± 11.30 | 0.002^a^ |
| Gender |  |  |  | <0.001^b^ |
| Male | 264(45.44) | 56 (60.87) | 55(72.37) |  |
| Female | 317(54.56) | 36(39.13) | 21(27.63) |  |
| ALT (U/L) |  |  |  | <0.001^c^ |
| <40 | 558(98.24) | 84(91.30) | 60(78.95) |  |
| ≥40 | 10(1.76) | 8(8.70) | 16(21.05) |  |
| AST (U/L) |  |  |  |  |
| <40 | 542(95.42) | 79(94.05) | 58(86.57) | 0.017^c^ |
| ≥40 | 26(4.58) | 5(5.95) | 9(13.43) |  |
| HCV genotype |  |  |  | 0.023^b^ |
| 1 | -- | 42(45.65) | 47(61.84) |  |
| Non-1 | -- | 28(30.43) | 10(13.16) |  |
| Mixed | -- | 22(23.91) | 19(25.00) |  |
| *IL28B*-rs12979860 |  |  |  | 0.807^b^ |
| CC | 519 (89.48) | 83 (91.21) | 67(88.16) |  |
| CT/TT | 61 (10.52) | 8 (8.79) | 9(11.84) |  |
| *IL28B*-rs8099917 |  |  |  | 0.694^b^ |
| TT | 488 (85.92) | 82 (89.13) | 65 (85.53) |  |
| TG/GG | 80 (14.08) | 10 (10.87) | 11(14.47) |  |

Group A: uninfected control cases; Group B: spontaneous clearance subjects; Group C: persistent infection patients.

Abbreviations: HCV, hepatitis C virus; SD, standard deviation; ALT, alanine transaminase; AST, aspartate transaminase; HD, hemodialysis patients; IVDU, Intravenous drug user.

Non-1 means genotype 2 and 3; Mixed means genotype1/2, 1/3, 2/3 and 1/2/3.

^a^*P* value of Kruskal-Wallis test among three/two groups.

^b^*P* value of *χ^2^*-test among three/two groups.

^c^*P* value of exact probability method among three/two groups.

**Supplementary Table 2. Demographic and clinical characteristics among HCV control, spontaneous clearance and persistent infection groups in IVDU patients.**

| Variables | Group A (%) | Group B (%) | Group C (%) | *P* |
| --- | --- | --- | --- | --- |
|  | n=797 | n=215 | n=141 |  |
| Age (mean ± SD) | 36.86 ± 8.72 | 35.64 ± 8.35 | 33.96 ± 6.27 | 0.001^a^ |
| Gender |  |  |  | <0.001^b^ |
| Male | 739(92.72) | 162 (75.35) | 99(70.21) |  |
| Female | 58(7.28) | 53(24.65) | 42(29.79) |  |
| ALT (U/L) |  |  |  | <0.001^b^ |
| <40 | 651(81.68) | 137(63.72) | 87(61.70) |  |
| ≥40 | 146(18.32) | 78(36.28) | 54(38.30) |  |
| AST (U/L) |  |  |  |  |
| <40 | 752(94.47) | 163(78.81) | 100(72.92) | <0.001^b^ |
| ≥40 | 44(5.53) | 52(24.19) | 41(19.08) |  |
| HCV genotype |  |  |  | <0.001^b^ |
| 1 | -- | -- | 89(64.03) |  |
| Non-1 | -- | 45(72.58) | 21(15.11) |  |
| Mixed | -- | 17(27.42) | 29(20.86) |  |
| *IL28B*-rs12979860 |  |  |  | 0.147^b^ |
| CC | 644 (80.80) | 180 (83.72) | 123 (87.23) |  |
| CT/TT | 153 (19.20) | 35 (16.28) | 18(12.77) |  |
| *IL28B*-rs8099917 |  |  |  | <0.001^b^ |
| TT | 500 (66.76) | 177 (82.33) | 126 (90.00) |  |
| TG/GG | 249 (33.24) | 38 (17.67) | 14 (10.00) |  |

Group A: uninfected control cases; Group B: spontaneous clearance subjects; Group C: persistent infection patients.

Abbreviations: HCV, hepatitis C virus; SD, standard deviation; ALT, alanine transaminase; AST, aspartate transaminase; HD, hemodialysis patients; IVDU, Intravenous drug user.

Non-1 means genotype 2 and 3; Mixed means genotype1/2, 1/3, 2/3 and 1/2/3.

^a^*P* value of Kruskal-Wallis test among three/two groups.

^b^*P* value of *χ^2^*-test among three/two groups.

**Supplementary Table 3.** Probes and primers of investigated *KIR*, *HLA- I* and *IL28B* SNPs for TaqMan assay.

| SNPs (Allele) | Gene | Region | MAF^a/b^ | *P^c^* | TaqMan-MGB probe/primers sequences (5’-3’) |
| --- | --- | --- | --- | --- | --- |
| rs35440472 | *KIR2DS4* | Intron Variant | 0.296/0.704 | 0.052 | Probe-A: VIC-ACACACAGAGAATAC-MGB |
| (G>A) |  |  |  |  | Probe-G: FAM-AACACACAGAGGATAC-MGB |
|  |  |  |  |  | Forward primer: CCGTCACTCCAGGGAGACA |
|  |  |  |  |  | Reverse primer: CACATTGAAATGCAGGCTTCTG |
| rs1130838 | *HLA-C* | Missense Variant | 0.187/0.813 | 0.491 | Probe-G: FAM-CTCATCGCTTGTAAAG-MGB |
| (G>A) |  |  |  |  | Probe-A: VIC-TCTCTCATCACTTGTAAAG-MGB |
|  |  |  |  |  | Forward primer: TTCCTCTTGTCCCACATCTCCT |
|  |  |  |  |  | Reverse primer: TCGAAACGTCCCAATCAAAG |
| rs2524094 | *HLA-C* | 2KB Upstream Variant | 0.756/0.244 | 0.052 | Probe-T: FAM-AGTGGAGAATCCTCAAC -MGB |
| (A>G) |  |  |  |  | Probe-C: VIC-AGTGGAGAATCCCCA-MGB |
|  |  |  |  |  | Forward primer: CAGGT TGGGAGAAGTGAAACTCA |
|  |  |  |  |  | Reverse primer: CAAGGGCCGTGTCTGCA |
| rs2308557 | HLA-C | Missense Variant | 0.140/0.860 | 0.113 | Probe-A: FAM-CGAGTGAACCTGCG-MGB |
| (G>A) |  |  |  |  | Probe-G: VIC-CGAGTGAGCCTGC-MGB |
|  |  |  |  |  | Forward primer: GAGTATTGGGACCGGGAGACACAGAA |
|  |  |  |  |  | Reverse primer: CCTCGCTCTGGTTGTAGTAGCC |
| rs12979860 | *IL28B* | Intron Variant | 0.900/0.100 | 0.122 | Probe-T: FAM-TCCCCGAAGGGTGA-MGB |
| (C>T) |  |  |  |  | Probe-C: VIC-CGAAGGCGCGAAC-MGB |
|  |  |  |  |  | Forward primer: TGCCTGTCGTGTACTGAACCA |
|  |  |  |  |  | Reverse primer: GAGCGCGGAGTGCAATTC |
| rs8099917 | *IL28B* | 5'UTR | 0.098/0.902 | 0.050 | Probe-T: FAM-TGAGCAATTTCACC-MGB |
| (T>G) |  |  |  |  | Probe-G: VIC-TGAGCAATGTCACCC-MGB |
|  |  |  |  |  | Forward primer: CAATTTGTCACTGTTCCTCCTTTTG |
|  |  |  |  |  | Reverse primer: TAAAGATGTGGGAGAATGCAAATGA |

Abbreviations: KIR, killer cell immunoglobulin-like receptor; HLA-I, human leukocyte antigen class I, IL28B, Interleukin 28B; HLA-C, human leukocyte antigen class I, C; SNPs, single nucleotide polymorphisms; MAF, minor allele frequency.

^a^ minor allele frequencies in control group (Group A).

^b^ minor allele frequencies from HapMap of Han Chinese in Beijing, China (CHB) or East Asia (EAS). (available at <https://www.ncbi.nlm.nih.gov/snp> ).

^c^*P* value of Hardy-Weinberg equilibrium for SNPs among control group (Group A).

**Supplementary Table 4.** Stratified analysis of the association of rs35440472 and rs1130838 with HCV susceptibility.

| SNPs | Subgroups | Group A | Group B | Group C | OR(95%CI)^a^ | *P*^a^ |
| --- | --- | --- | --- | --- | --- | --- |
|  |  | n (GG/GA/AA) | n (GG/GA/AA) | n (GG/GA/AA) |  |  |
| rs35440472 | Age |  |  |  |  |  |
|  | <50 | 354/409/135 | 74/100/54 | 48/95/40 | **1.417 (1.181-1.699)** | **＜0.001** |
|  | ≥50 | 125/159/71 | 23/36/20 | 9/18/7 | 1.249 (0.905-1.724) | 0.037 |
|  | Gender |  |  |  |  |  |
|  | Male | 342/394/142 | 70/94/54 | 38/85/31 | **1.378(1.144-1.659)** | **0.001** |
|  | Female | 137/174/64 | 27/42/20 | 19/28/16 | 1.204(0.875-1.656) | 0.254 |
|  | ALT (U/L) |  |  |  |  |  |
|  | <40 | 433/506/180 | 67/107/47 | 42/75/30 | **1.348(1.135-1.602)** | **0.001** |
|  | ≥40 | 41/57/23 | 30/29/27 | 15/38/17 | **1.503(1.028-2.197)** | **0.035** |
|  | AST (U/L) |  |  |  |  |  |
|  | <40 | 455/537/192 | 77/111/55 | 41/84/33 | **1.392(1.180-1.641)** | **＜0.001** |
|  | ≥40 | 19/25/11 | 20/19/17 | 13/23/14 | 1.131(0.697-1.835) | 0.697 |
|  | Route of infection |  |  |  |  |  |
|  | HD | 209 /270/102 | 20/50/22 | 23/44/9 | 1.191(0.909-1.561) | 0.206 |
|  | IVDU | 270/298/104 | 77/86/52 | 34/69/38 | **1.399(1.145-1.710)** | **0.001** |
| rs1130838 | Age | n (GG/GA/AA) | n (GG/GA/AA) | n (GG/GA/AA) |  |  |
|  | <50 | 721/250/30 | 157/66/5 | 136/36/11 | 1.259(0.602-2.635) | 0.540 |
|  | ≥50 | 269/98/10 | 53/20/6 | 19/12/3 | **4.542(1.492-13.830)** | **0.008** |
|  | Gender |  |  |  |  |  |
|  | Male | 719/257/27 | 151/60/7 | 110/37/7 | 1.703(0.824-3.521) | 0.151 |
|  | Female | 271/91/13 | 59/26/4 | 45/11/7 | 2.526(0.779-8.188) | 0.122 |
|  | ALT (U/L) |  |  |  |  |  |
|  | <40 | 874/316/30 | 155/60/6 | 104/31/12 | **2.397(1.261-4.557)** | **0.008** |
|  | ≥40 | 109/31/5 | 55/26/5 | 51/17/2 | 1.202(0.302-4.781) | 0.794 |
|  | AST (U/L) |  |  |  |  |  |
|  | <40 | 937/331/32 | 172/63/8 | 110/37/11 | 2.363(1.277-4.373) | 0.006 |
|  | ≥40 | 44/16/4 | 32/22/2 | 37/11/2 | 0.765(0.133-4.411) | 0.765 |
|  | Route of infection |  |  |  |  |  |
|  | HD | 413/151/17 | 60/26/6 | 54/16/6 | **4.027(1.534-10.576)** | **0.005** |
|  | IVDU | 577/197/23 | 150/60/5 | 101/32/8 | 1.076(0.478-2.422) | 0.859 |

Abbreviations: HCV, hepatitis C virus; OR, odds ratio; CI, confidence interval; HD, hemodialysis patients; IVDU, Intravenous drug user.

Group A: uninfected control cases; Group B: spontaneous clearance subjects; Group C: persistent infection patients; Group (B+C): Infected individuals.

^a^ *P* value, OR and 95% CIs of Group A versus Group (B+C) were calculated based on the logistic regression model, adjusted by gender, age, ALT, AST, *IL28B*-rs12979860, *IL28B-*rs8099917 and route of infection.

Bold type indicates statistically significant results.

**Supplementary Table 5.** Annotation of variants with strong linkage disequilibrium with SNP rs1130838 in HaploRegV4.1.

| Chr | Pos  (hg19) | LD  (𝑟2) | Variant | Ref | Alt | ASN freq | Enhancer histone marks | DNAse | Proteins bound | Selected eQTL hits | Motifs changed | dbSNP func annot |
| --- | --- | --- | --- | --- | --- | --- | --- | --- | --- | --- | --- | --- |
| 6 | 31269347 | 1.00 | rs1130838 | T | C | 0.81 | 9 tissues | BLD,BLD,BLD |  | 156 hits | PLZF | missense |
| 6 | 31269385 | 1.00 | rs35708511 | C | G | 0.81 | 9 tissues | BLD,BLD |  | 156 hits | INSM1,p300 | missense |
| 6 | 31269546 | 1.00 | rs68094471 | A | G | 0.81 | 11 tissues | BLD |  | 156 hits |  | intronic |
| 6 | 31269576 | 1.00 | rs67827555 | T | C | 0.81 | 11 tissues | BLD,BLD,BLD |  | 157 hits | 4 altered motifs | intronic |
| 6 | 31269628 | 1.00 | rs56010430 | C | T | 0.81 | 9 tissues | 4 tissues |  | 156 hits | SREBP | intronic |
| 6 | 31269660 | 1.00 | rs66459704 | T | C | 0.81 | 9 tissues | 4 tissues | POL2 | 156 hits | Hsf,YY1 | intronic |
| 6 | 31269686 | 1.00 | rs66620546 | C | G | 0.81 | 9 tissues | BLD | POL2 | 156 hits | Myf,Zbtb12 | intronic |
| 6 | 31269786 | 1.00 | rs3819287 | C | T | 0.81 | 9 tissues | BLD,BLD | POL2,  POL24H8 | 159 hits | T3R | intronic |
| 6 | 31269815 | 1.00 | rs9264606 | A | C | 0.81 | 9 tissues | BLD,BLD,BLD | POL2,  POL24H8 | 163 hits | PU.1,Sox | intronic |
| 6 | 31269883 | 1.00 | rs9264608 | A | C,G,T | 0.81 | 12 tissues | 5 tissues | POL2,  POL24H8 | 158 hits |  | intronic |
| 6 | 31269887 | 1.00 | rs9264609 | C | T | 0.81 | 12 tissues | BLD,BLD,SKIN | POL2,  POL24H8 | 156 hits | SP1,TATA,THAP1 | intronic |

Chr: chromosome, Pos: position, LD: linkage disequilibrium in Asian population, Ref: reference, Alt: alternative, freq: frequency.

## Supplementary Figures

**Supplementary Figure 1.** The representative result of a TaqMan allelic discrimination assay ( rs35440472).


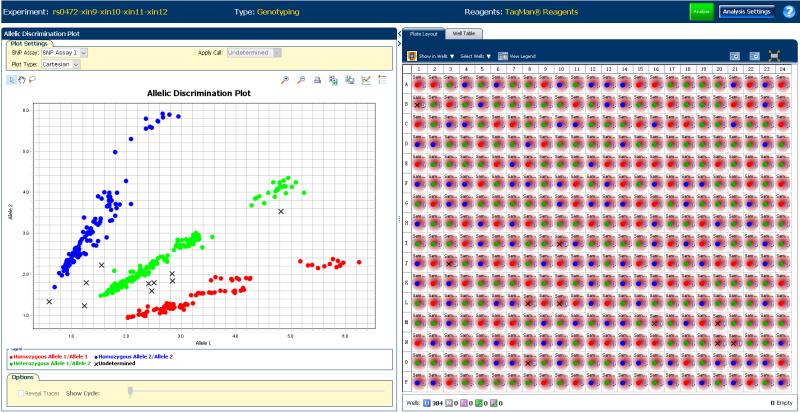


The blue points present rs35440472-GG; the green points present rs35440472-GA; the red points present rs35440472-AA; the fork signs present failed genotyping.

**Supplementary Figure 2.** The eQTLs of rs35440472 for *KIR2DS4* expression in the liver tissue.


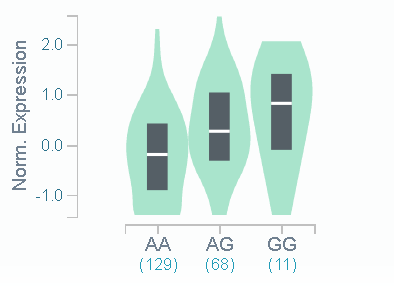


Data available: http://genome.ucsc.edu/

**Supplementary Figure 3.** Functional annotation for rs1130838 using ENCODE data from UCSC genome browser.


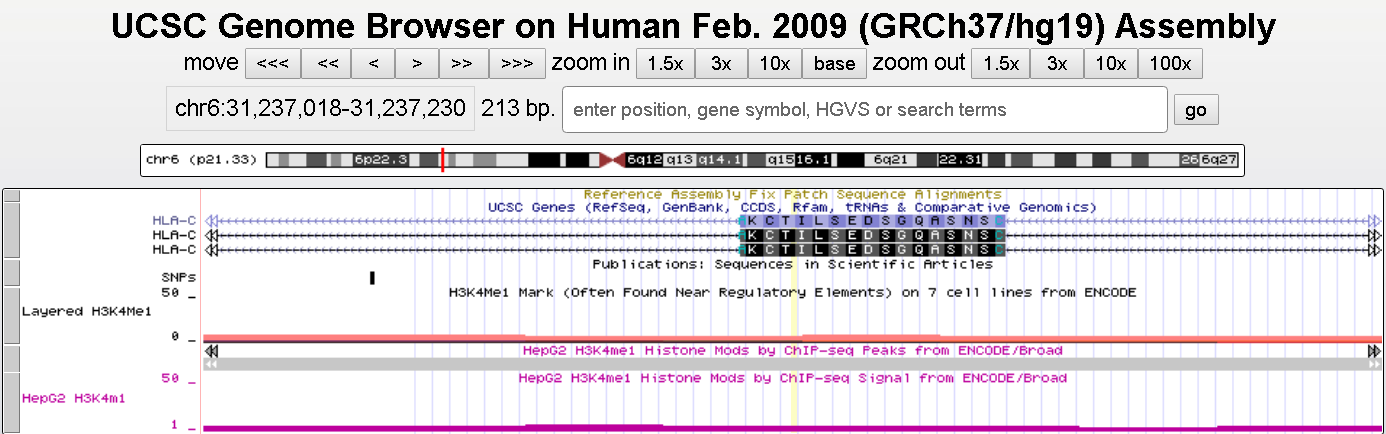


The histone H3 at lysine 4 (H3K4Me1) marker in 7 cell lines (GM12878, H1-hESC, HSMM, HUVEC, K562, NHEK, and NHLF cells) as well as HepG2 liver cell line are presented. The light blue line indicates the position of SNP rs1130838 (Data available: <http://genome.ucsc.edu/>).
